# ECG Adapted Fastest Route Algorithm to Localize the Ectopic Excitation Origin in CRT Patients

**DOI:** 10.3389/fphys.2019.00183

**Published:** 2019-03-11

**Authors:** Danila Potyagaylo, Mikhail Chmelevsky, Peter van Dam, Margarita Budanova, Stepan Zubarev, Tatjana Treshkur, Dmitry Lebedev

**Affiliations:** ^1^EP Solutions SA, Yverdon-les-Bains, Switzerland; ^2^Almazov National Medical Research Center, Saint Petersburg, Russia; ^3^Cardiology Department, University Medical Center Utrecht, Utrecht, Netherlands

**Keywords:** ECG imaging, fastest route algorithm, FRA, dynamic time warping, inverse problem of ECG, CRT, inhomogeneous excitation propagation

## Abstract

Although model-based solution strategies for the ECGI were reported to deliver promising clinical results, they strongly rely on some a priori assumptions, which do not hold true for many pathological cases. The fastest route algorithm (FRA) is a well-established method for noninvasive imaging of ectopic activities. It generates test activation sequences on the heart and compares the corresponding test body surface potential maps (BSPMs) to the measured ones. The test excitation propagation patterns are constructed under the assumption of a global conduction velocity in the heart, which is violated in the cardiac resynchronization (CRT) patients suffering from conduction disturbances. In the present work, we propose to apply dynamic time warping (DTW) to the test and measured ECGs before measuring their similarity. The warping step is a non-linear pattern matching that compensates for local delays in the temporal sequences, thus accounting for the inhomogeneous excitation propagation, while aligning them in an optimal way with respect to a distance function. To evaluate benefits of the temporal warping for FRA-based BSPMs, we considered three scenarios. In the first setting, a simplified simulation example was constructed to illustrate the temporal warping and display the resulting distance map. Then, we applied the proposed method to eight BSPMs produced by realistic ectopic activation sequences and compared its performance to FRA. Finally, we assessed localization accuracy of both techniques in ten CRT patients. For each patient, we noninvasively imaged two paced ECGs: from left and right ventricular implanted leads. In all scenarios, FRA-DTW outperformed FRA in terms of LEs. For the clinical cases, the median (25–75% range) distance errors were reduced from 16 (8–23)mm to 5 (2–10)mm for all pacings, from 15 (11–25)mm to 8 (3–13)mm in the left, and from 19 (6–23)mm to 4 (2–8)mm in the right ventricle, respectively. The obtained results suggest the ability of temporal ECG warping to compensate for an inhomogeneous conduction profile, while retaining computational efficiency intrinsic to FRA.

## 1. Introduction

Due to the aging population, increase in unhealthy lifestyle and advances in acute management of myocardial infarction, heart failure is becoming the world leading cause of death. Thereby congenital and acquired ventricular dysfunction result in poor short- and mid-term prognosis, making cardiac resynchronization therapy (CRT) the first choice of care to decrease hospitalization and improve the quality of life for heart failure patients. However, around 30% of CRT candidates fail to respond to this treatment, which leads to increasing morbidity and involved medical insurance costs (Daubert et al., 2016). In general, patient-specific optimization of CRT treatment and selection, being essential for improved success rates, can be accomplished only upon knowledge of underlying cardiac substrate and electrophysiological properties.

Invasive acquisition of individual heart model parameters is laborious, associated with risks and, therefore, prohibitive for many CRT candidates. For these candidates, the ECG imaging (ECGI) technique represents a noninvasive alternative (Gulrajani, 1998; Pullan et al., 2001). Based on a patient-specific geometry, ECGI maps measured body surface potentials to activation times on the cardiac anatomy. Although there has recently been a distinct interest raise from both engineering and clinical communities, this technology has not yet found its niche in the clinical work-flow (Cluitmans et al., 2018). For this, known technical issues have to be solved and clear clinical benefits have to be defined in cooperation with physicians. Furthermore, the validation of ECGI is extremely challenging, which is mostly due to the lack of invasively obtained high quality data.

In a nutshell, ECG imaging consists in solving an ill-posed problem of finding cardiac sources configurations causing the observed body surface potential maps (BSPMs) (Cluitmans et al., 2018). Different approaches to ECGI, or inverse problem of ECG, could deliver information on the earliest activation site (Erem et al., 2014a; van Dam et al., 2016; Giffard-Roisin et al., 2017; Yu et al., 2018), isochronal, isopotential, or phase maps (van Dam et al., 2009a; Revishvili et al., 2015; Wang et al., 2016; Rodrigo et al., 2017), and substrate characterization (Rudy, 2013; Sohns et al., 2018). One way to tackle the inverse problem is the classical regularization by imposing appropriate regularization constraints (Brooks et al., 1999). Another, model-based, approach consists in employing a realistic excitation propagation model and fitting the model parameters to match the measured BSPMs. In van Dam et al. (2009a) the nonlinear inverse problem was solved based on the action potential wave forms specified by two parameters at each cardiac node, activation and recovery times. The initial estimation for this task was provided by a physiologically inspired fastest route algorithm. Wang et al. developed a Bayesian framework for coupling personal data with the prior model based on the unscented Kalman filter for integration of the nonlinear action potential's dynamics (Wang et al., 2011). Performance of an artificial network optimizing cellular-automaton excitation parameters in a 3-D heart was presented in Li and He (2001) and Liu et al. (2008). Parameter tuning in a more complex bidomain model was evaluated in terms of simulated ECG similarities with the measured 12-lead signals in heart-failure patients (Potse et al., 2014). Dhamala et al. (2017) introduced a computational framework featuring spatially adaptive coarse-to-fine optimization of cardiac excitation properties to match the measured ECGs. The work by Giffard-Roisin et al. (2017) aimed at noninvasive estimation of the global conduction velocity and activation onset by regressing the measured BSPMs from a simulated database.

Despite being one of the most straightforward among the existing model-based inverse strategies, the fastest route algorithm (FRA) has demonstrated a number of encouraging simulation as well as clinical results in imaging of ectopic and normal activation sequences (van Dam et al., 2009a, 2016; Oosterhoff et al., 2016; Janssen et al., 2018). For a patient-specific cardiac geometry, FRA simulates excitation patterns starting from every node of the discretized heart mesh. The obtained activation sequences are converted to the BSPMs by solving a linear forward problem of ECG for the corresponding volume conductor model. This is followed by a full-search step resulting in the activation sequence associated to the BSPMs with the highest correlation compared to the measured electrocardiograms. Depending on the clinical application, the best sequence can be either used independently, e.g., for estimation of the excitation origin (Potyagaylo, 2016; Potyagaylo et al., 2016a,b), or followed by an iterative nonlinear least-squares (NLLS) procedure (van Dam et al., 2009a; Erem et al., 2014b). The NLLS itself is a severely ill-posed optimization problem with multiple local minima, which makes it extremely sensitive to the initial estimate (Modre et al., 2002; Janssen et al., 2018).

For a global conduction velocity (CV), an initialization provided by FRA was shown to be robust with respect to the forward modeling errors in an *in silico* study in Potyagaylo et al. (2016a). For the calculation of the FRA activation sequences, transmural cardiac connections are assigned with half the value for propagation speed in the direction tangential to the heart surface (van Dam et al., 2009a). While this model aims at taking into account a slower transmural wave propagation, it can neither fully compensate for anisotropic excitation nor tackle differences in the local CVs due, for instance, to scar. Furthermore, the NLLS optimization step was demonstrated to be highly sensitive with respect to the assumed propagation velocity used within FRA (Erem et al., 2014b).

To overcome the above-mentioned limitations of the standard FRA approach, we propose to apply dynamic time warping (DTW) to the BSPMs. The simulated BSPMs are adjusted and aligned with the measured signals. After the alignment, euclidean “distances” between warped simulated test potentials and recorded BSPMs are calculated. The cardiac mesh node associated with the excitation pattern corresponding to the smallest error is considered the sought-after activation origin. In the sequel, we denote this method as FRA-DTW.

## 2. Materials and Methods

To demonstrate superior performance of the proposed strategy, we first consider a simplified focal excitation scenario with an artificially introduced region of slow CV and provide an ECG signal warping example. Then, we compare performance of FRA-DTW against the standard correlation-based FRA for eight realistic simulation cases of ectopic excitation used in Janssen et al. (2018). We analyze localization errors (LEs) and visualize both correlation and DTW-based distance maps for studying possible reconstruction ambiguities.

Finally, we performed clinical evaluation and comparison of both techniques using isolated univentricular left and right ventricular (RV and LV, respectively) pacing in 10 patients (*n* = 10) with previously implanted CRT devices. For the first time, we quantitatively estimate performance of FRA and time warping applied to FRA-generated BSPMs on the CRT patients.

### 2.1. Source Model and Fastest Route Algorithm (FRA)

In this study equivalent dipole layer (EDL) is used. For equal anisotropy ratios in intra- and extracellular electrical conductivity tensors the cardiac current sources were shown to behave like an EDL (Geselowitz and Miller, 1983; Yamashita and Geselowitz, 1985; Geselowitz, 1989). The EDL has an orientation normal to the heart surface, encompassing both endo- and epicardium, and is proportional to the surface transmembrane potentials (TMP) (van Oosterom and Jacquemet, 2005; van Dam et al., 2009a,b).

Furthermore, this source model allows a linear relationship between the TMP and BSPMs given by a transfer (also known as forward, or lead-field) matrix *A*, which depends solely on the volume conductor model. For the depolarization phase, when the cardiac cells can be assumed to be either at rest or activated, electrical activity of the heart is fully described by the activation times $\tau \left(\vec{x}\right)$. Then, the expression for body surface potentials at time *t* reads as follows (Huiskamp and Van Oosterom, 1988; Janssen et al., 2018):

(1) $$y(t)=\int_{S_{h}}H(t-\tau (\vec{x}))A(\vec{x})d\vec{x}$$

where $A\left(\vec{x}\right)$ is the lead-field for $\vec{x}$, i.e., the potentials generated by an infinitesimal source at location $\vec{x}$ on the heart surface *dS*_*h*_, and *H*(*t*) is the Heaviside step function characterizing “on” and “off” states of the cellular activity. For the present work, the transfer matrix *A* was calculated by means of the boundary element method (BEM).

The inverse problem of ECG associated with (1) consists in finding the depolarization (activation) times $\tau \left(\vec{x}\right)$ on the heart surface. Due to its intrinsic ill-posedness, this nonlinear optimization problem has multiple local minima and is, therefore, highly sensitive to the initial estimate (Modre et al., 2002; Erem et al., 2014b). With this respect, the fastest route algorithm (FRA) was reported to provide a physiologically meaningful initialization for (1) (van Dam et al., 2009a). In essence, FRA is a, possibly multi-foci, search, where each cardiac node is considered as an initial focus. For computation of the corresponding test activation sequences, a times matrix *T* based on the adjacency graph of the triangulated heart mesh is used. Although a global conduction velocity is assumed for calculation of *T*, the transmural wavefront speed is set to be twice less than those along the heart surface, which mimics cardiac transmural anisotropy. For each cardiac node, the respective BSPMs are compared to the measured signals on the basis of correlation coefficient (CC), providing a correlation map on the heart surface. The sequence resulting in the highest correlation is taken as the initialization for (1). However, the best activation pattern can be effectively used together with the accompanying correlation map in order to estimate the solution uncertainty and illustrate reconstruction ambiguities (Potyagaylo et al., 2016a; Janssen et al., 2018).

Despite its simplicity, FRA has proven to be a robust method delivering a physiologically meaningful solution approximation for (multi-foci) excitation patterns (Oosterhoff et al., 2016; Potyagaylo et al., 2016a; van Dam et al., 2016). Nonetheless, FRA gets computational very expensive when it accounts for regions with a local different CV. Consequently information on their anatomical location needs to be incorporated explicitly into the activation model given by the matrix *T*. In these cases, FRA scales the global CV (0.8 m/s for this work) in order to match the QRS complex duration, which can apparently result in a distorted CC distribution and a misleading solution. To alleviate this FRA drawback, we propose to apply temporal warping to the test and measured BSPMs before calculating their mismatch.

### 2.2. Dynamic Time Warping (DTW)

Dynamic time warping (DTW) is an algorithm for measuring similarity between time series that may vary in velocity, even if there were acceleration or deceleration phases in one of the signals. We hypothesized that local CV differences reduces the accuracy of FRA performance, which can at least be partially compensated by the nonlinear time warping of the simulated BSPMs in the FRA-DTW method.

Within the FRA formulation all test activation sequences are linearly temporally scaled to match the reference BSPMs duration. This results in a particular case for the dynamic time warping, where both reference *Y* and test $\tilde{Y}$ ECG sequences have the same length of *T* ms, i.e., $\tilde{Y},Y\in {\mathbb{R}}^{P\times T}$ with *P* being the number of electrodes. Outlining the general approach, a local distance measure $c\left(\tilde{y},y\right)$ between their elements is introduced first. Each element represents body surface potentials for one time instance recorded at *P* positions. In this way, we align the whole temporal BSPMs matrices simultaneously for all electrodes positions. Then, a cost matrix *C* ∈ ℝ^*T* × *T*^ is constructed by local costs for all element pairs from $\tilde{Y}$ and *Y*. Provided *C*, the goal of the DTW algorithm is to find an optimal temporal alignment between $\tilde{Y}$ and *Y*, i.e., such an alignment that runs through the two-dimensional matrix *C* along the path of the lowest total cost. In other words, DTW minimizes the body surface potentials mismatch by a proper reordering of the temporal indices.

More formally, a warping path *p* = (*p*_1_, …, *p*_*L*_) with *p*_*l*_ = (*n*_*l*_, *m*_*l*_) ∈ [1, *T*] × [1, *T*] and *l* ∈ [1, *L*] is defined by assigning the elements ${\tilde{y}}_{n_{l}}$ in $\tilde{Y}$ to the elements *y*_*m*_*l*__ in *Y*. While *n*_*l*_ and *m*_*l*_ take the values of temporal indices, *L* denotes the number of path elements which is in general greater than the sequences' length *T*. This is the case when at least one element in one sequence is matched to multiple elements in the other sequence (Müller, 2007).

Furthermore, a feasible warping path is specified to satisfy some common sense observations: boundary, monotonicity and step size conditions have to be met. The boundary condition means that the first index from the first sequence must be aligned with the first index of the second sequence (and possibly following indices), i.e., *p*_1_ = (1, 1). Furthermore, the last index of the first sequence must be aligned with the last index of the second one (and possibly previous indices), i.e., *p*_*L*_ = (*T, T*). The monotonicity condition applies to both positional arguments of *p*: *n*_1_ ≤ *n*_2_ ≤ … ≤ *n*_*L*_ and *m*_1_ ≤ *m*_2_ ≤ … ≤ *m*_*L*_ and reflects the requirement of a proper time progression. The third condition restricts the step size in each index: *p*_*l*+1_ − *p*_*l*_ ∈ (1, 0), (0, 1), (1, 1) for *l* ∈ [1, *L* − 1], meaning that every index in both arrays must get a pair from the other sequence.

Under these conditions, the total cost function $c_{p}\left(\tilde{Y},Y\right)$ is formed by a sum of distances between elements from $\tilde{Y}$ and *Y* with the path indices (*n*_*l*_, *m*_*l*_):

(2) $$c_{p}(\tilde{Y},Y)=\sum_{l=1}^{L}c({\tilde{y}}_{n_{l}},y_{m_{l}})=\sum_{l=1}^{L}c(\tilde{Y}(:,n_{l}),Y(:,m_{l}))$$

and the DTW algorithm minimizes the total cost among all feasible paths (Müller, 2007):

(3) $$c_{p^{*}}(\tilde{Y},Y)=\min\{c_{p}(\tilde{Y},Y)|p\text{ is a warping path}\}$$

Obviously, the number of all possible paths *c*_*p*_ through a two-dimensional grid *C* is very large. In order to reduce the computational complexity, we used a dynamic programming algorithm for calculating the optimal path p^*^. For this purpose, the accumulated cost matrix *D* ∈ ℝ^*T* × *T*^ is introduced as follows:

(4) $$D(n,m)=c_{p^{*}}(\tilde{Y}(:,1:n),Y(:,1:m))$$

The matrix *D* contains optimal costs for all temporal subsequences in $\tilde{Y}$ and *Y* and its element *D*(*T, T*) is equal to the optimal cost function value $c_{p^{*}}\left(\tilde{Y},Y\right)$. Furthermore, it can be shown that the matrix elements satisfy the following identity (Müller, 2007):

(5) $$\begin{array}{l} D(n,m)=\min\{D(n-1,m-1),D(n-1,m),D(n,m-1)\}\\ \;+c({\tilde{y}}_{n},y_{m}) \end{array}$$

Extending the matrix by an additional row and column and setting *D*(0, :) = *D*(:, 0) = ∞ and *D*(0, 0) = 0 facilitates recursive calculation of *D*.

Provided the accumulated cost matrix *D*, an optimal warping path $p^{*}=\left(p_{1},\ldots ,p_{L}\right)$ is computed in the reverse manner by starting from the index *p*_*L*_ = (*T, T*):

(6) $$p_{l-1}=\{\begin{array}{c} \begin{array}{cc} (1,m-1),\; & \text{if }n=1 \end{array}\\ \begin{array}{cc} (n-1,1),\; & \text{if }m=1 \end{array}\\ \text{arg min}\{D(n-1,m-1),\;\\ \begin{array}{cc} \;D(n-1,m),D(n,m-1)\} & \text{otherwise} \end{array} \end{array}$$

Apparently, the optimal warping path depends on the choice of a cost function $c\left({\tilde{y}}_{n_{l}},y_{m_{l}}\right)$ being the only algorithm's parameter. For this study, we used the euclidean norm as the distance function, i.e., $c\left({\tilde{y}}_{n_{l}},y_{m_{l}}\right)=||{\tilde{y}}_{n_{l}}-{\tilde{y}}_{m_{l}}|{|}_{L_{2}}$. Similar to Giffard-Roisin et al. (2018), both test and measured BSPMs signals were normalized beforehand in order to reduce the influence of torso inhomogeneities on the ECG amplitude. To this end, we scaled all BSPMs signals column-wise by subtracting the mean and component-wise scaling to unit variance (preprocessing scale function from sciki-learn (Pedregosa et al., 2011) was used). For a pseudo-code of the employed inverse pipeline the reader is referred to the Appendix A.

Same as within standard FRA methodology, for each cardiac node we computed the corresponding test activation sequence and the associated test BSPMs. Then, each test BSPMs sequence in pair with the reference BSPMs were temporally warped by computing the optimal path cost (3). As minimizing the cost function *c*_*p*_ in (3) is equivalent to maximization of 1/*c*_*p*_, for the sake of consistency with the standard FRA approach searching for the maximum correlation, we used the reciprocal (or inverse) distance function 1/*c*_*p*_ for visualization. Calculated for test BSPMs relating to each cardiac node, the obtained reciprocal distances 1/*c*_*p*_ can be displayed on the heart surface. By analogy with the FRA-based correlation maps, the resulting inverse distance maps can be employed as an uncertainty quantification tool.

#### 2.2.1. Simplified Simulation Case of Slow CV Area

**Figure 1:**
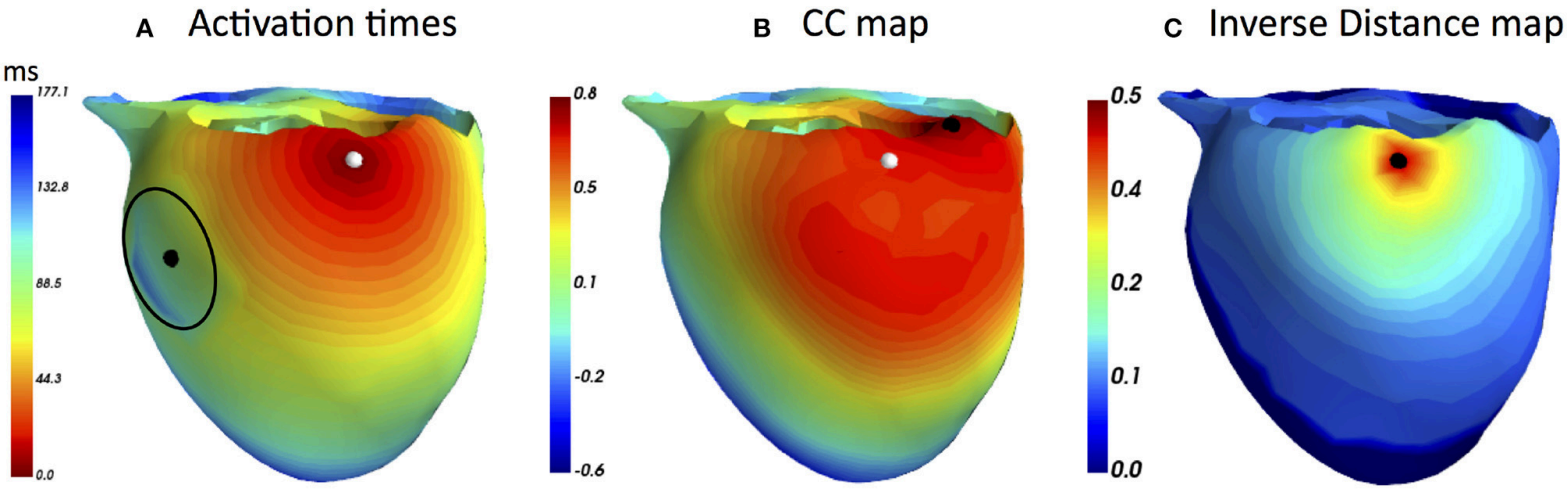
**(A)** Modified ectopic excitation sequence starting from the node marked by the white marker with the reduced CV in the area centered at the black dot and enclosed by the black line. **(B)** Correlation map generated by the standard FRA with the reconstructed onset marked by the black point. It is worth noting that a relatively large area exhibits upper percentile of the high correlation coefficient values, implying higher solution ambiguity and, thereby, weakening the prediction **(C)** Distribution of the DTW-based inverse distance function (dimensionless due to the ECG scaling). The black point overlaps the reference origin, meaning that the temporal warping could account for the excitation delay and resulted in the exact inverse localization. Notably, only a few heart nodes corresponded to the upper percentile of the inverse distance.

First, we provide a simulation example based on a realistic human geometry. For the considered heart mesh, the activation times matrix *T* was computed as utilized by FRA. Next, a node on the lateral LV wall was selected to be the ectopic focus, the one to be noninvasively localized. Additionally, a region with 20 mm radius about 60 mm from the selected focus in which the CV was reduced by a factor of three. According to this modification in CV, the activation times were created, see Figure 1A. The corresponding BSPMs from this activation sequence was computed. For both FRA and FRA-DTW the unaltered times matrix *T* was used. For FRA the correlation map was used to estimate the focus, for FRA-DTW the reciprocal distance as similarity measure was used. In Figure 1B, the FRA-based correlation map is visualized together with the true and localized origins shifted by 31 mm in the direction opposite to the location of the slow CV area relative to the reference focus. In contrast to that, the nonlinear temporal warping was able to account for this modeling error, which is reflected in the exact onset reconstruction provided by the inverse cost map shown in Figure 1C.

**Figure 2:**
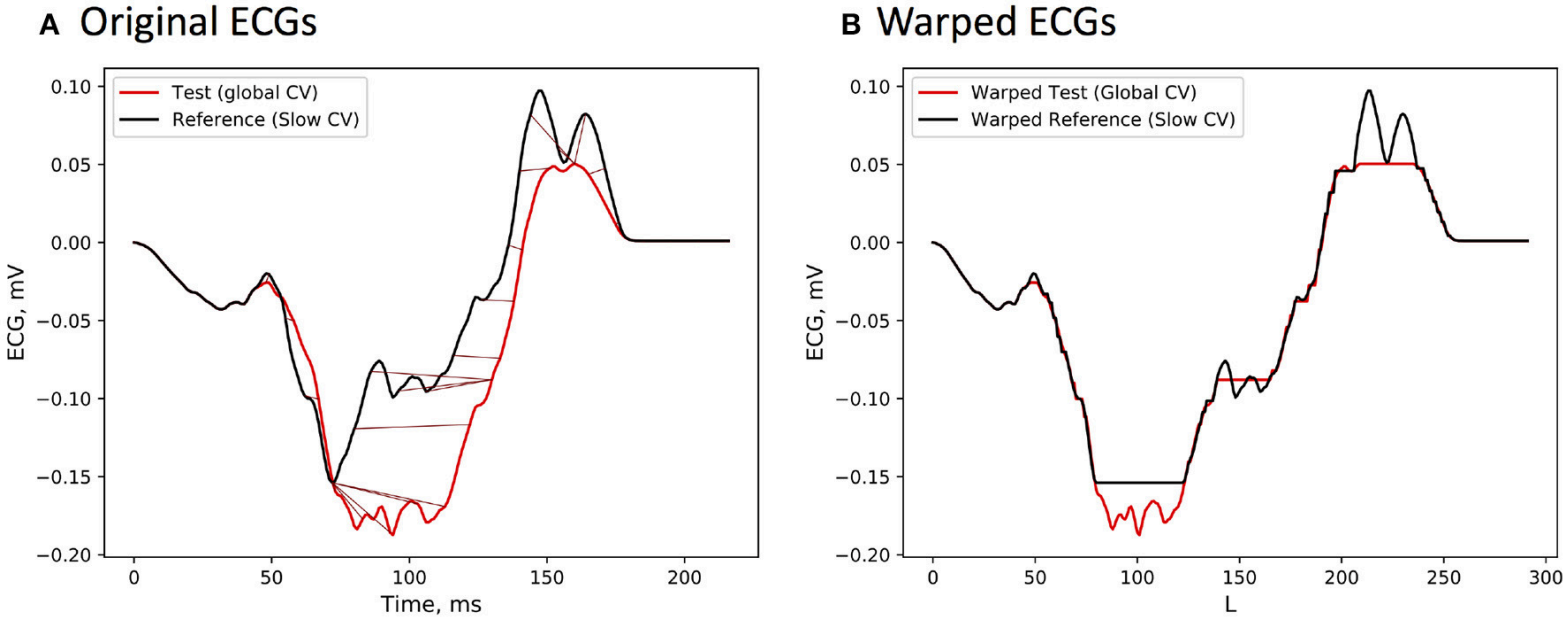
**(A)** ECG signals from the reference BSPMs, which was computed from the activation sequence with locally modified CV value, and the test BSPMs generated by the standard FRA under the assumption of a global CV. The signals differ only on the time interval corresponding to the wavefront passing through the low CV region. **(B)** Temporally warped ECG sequences with the path length *L*.

To illustrate temporal warping of the ECG signals, the ECG channel with the lowest correlation (88 %) is shown. Figure 2A depicts both reference and test signals together with their nonlinear alignment, whilst Figure 2B visualizes the optimally warped signals. As mentioned previously, the duration of an optimal warping path $c_{p^{*}}$ is generally larger than the signals' length due to the fact that multiple elements of one sequence can be aligned with the same element of the other one. Obviously, no temporal shifts can compensate for amplitudes mismatch due to a complex nonlinear relationship (1) connecting activation times on the heart to the body surface potentials. However, the difference in a local conduction speed was accounted for by the warping function.

### 2.3. Realistic Simulations of Ectopic Excitation

After the proof-of-concept provided by the manually constructed and, certainly, oversimplified simulation case, we conducted a comparison between FRA and FRA-DTW and evaluated performance of the latter for realistic excitation patterns. For this study eight ectopic activation sequences presented in Janssen et al. (2018) were used.

In short, the excitation propagation patterns were simulated with the monodomain model and the BSPMs were then generated for a realistic finite-element volume conductor with an anisotropic heart model. In Janssen et al. (2018), the authors investigated the influence of bidomain conductivity tensors in the forward modeling on the quality of inverse reconstructions obtained with the EDL model. In the present work, only the most realistic case is considered, the model with unequal anisotropy ratio in the intra- and extracellular spaces.

In Janssen et al. (2018) FRA was used to compute an initial estimate for solving the subsequent NLLS (1). As the final solution was shown to heavily depend on the initialization, the goal of this study was to determine, whether the FRA with incorporated temporal warping is able to provide an improved estimate compared to its standard version. The focus locations considered for this study listed: “two foci on both sides of the septal wall, two left ventricular free wall foci, two foci on the right ventricular free wall, and two beats originating from a basal part of the ventricles close to the septal wall” (Janssen et al., 2018).

### 2.4. Clinical Data

The implanted biventricular pacemaker leads position are exactly known from CT scans, providing ideal ECGI validation data for single paced activation sequences from the LV and RV for each patient. Therefore, we enrolled in this study 10 patients (*n* = 10) from 54 to 70 years (median 65; 25–75% range 59–64; 8 male) with previously implanted CRT devices. Among them nine patients had a left bundle branch block (LBBB) QRS morphology of the intrinsic rhythm, and seven of them had a LV scar with low conduction velocity zones after myocardial infarction. These LV zones were not taken into account in the inverse procedure.

The study was reviewed and approved by the Ethical Committee of Almazov National Medical Research Center in Saint Petersburg, Russia. Written informed consent was obtained from each patient after detailed description and explanation of the study before the procedures. This single-center cross-sectional study was performed in accordance with the Good Clinical Practice guidelines and Helsinki declaration for biomedical research.

#### 2.4.1. ECG and CT Data

A total maximum number of 240 body surface electrodes were applied on the patient's torso and connected to the multichannel Amycard 01C EP system ECG amplifier (EP Solutions SA, Switzerland). CRT device in each patient was programmed and continuous ECGs of isolated RV/LV pacing from implanted leads at rate not more than 90 bpm were recorded during 10 sec. The pacing amplitude and duration were selected individually based on the originally established parameters of the CRT device. The original parameters were set up 2–3 months prior to the procedure during a regular check-up based on standard criteria in the clinical practice. According to the results of an automatic threshold test in the CRT device, the minimal spike amplitude and duration have been selected to have stable effective capture during RV and LV pacing. All measurements were performed during breath hold. Immediately after recording of the multichannel ECG, all patients underwent cardiac CT imaging with applied body surface electrodes. The obtained CT data was imported into Amycard 01C EP system software in DICOM format to reconstruct polygonal meshes of the torso, lungs and detailed epi-endocardial ventricular heart models based on the semi-automatic segmentation.

#### 2.4.2. Anatomical Models

For our inverse calculations, a piece-wise heterogeneous volume conductor model was used with thorax, lungs and ventricular blood masses as regions with an electrical conductivity deviating from that of the torso. Following the guidelines commonly accepted in the ECGI community, the assigned electrical conductivity values were 0.6, 0.04 and 0.2 Sm/m for blood masses, lungs and ventricles, respectively (see e.g., Modre et al., 2002; van Dam et al., 2009a).

#### 2.4.3. Quality Metrics

In order to estimate the inverse routine performance, the distances were computed between the known pacemaker locations and noninvasively identified earliest excitation sites. All estimated LV and RV pacemaker lead positions were localized for the epi- or endocardial heart surfaces, respectively. The geodesic distance was considered as a more reliable quality measure for curved surfaces, and for endocardial solutions separated from a pacemaker by the septal wall. As a supplementary metric targeting the misclassified ectopic origins, we analyzed whether the overall earliest activation site was found on the same (endo- or epicardial) cardiac surface as the corresponding pacemaker lead.

In addition, we performed a bias-corrected and accelerated bootstrap analysis (in accordance to Efron and Tibshiran correction) in order to check the stability, variability, and robustness of the estimated ECGI accuracy and provide more confidence to the results of this study. We used 2.5 and 97.5 percentile interval for the calculation of the 95% confidence intervals for reference limits in all continuous variables. Bootstrap was performed with 1,000 replications for each variable with a Mersenne twister as a random number generator.

## 3. Results

### 3.1. ECGI for Realistic Simulations of Ectopic Excitation

**Figure 3:**
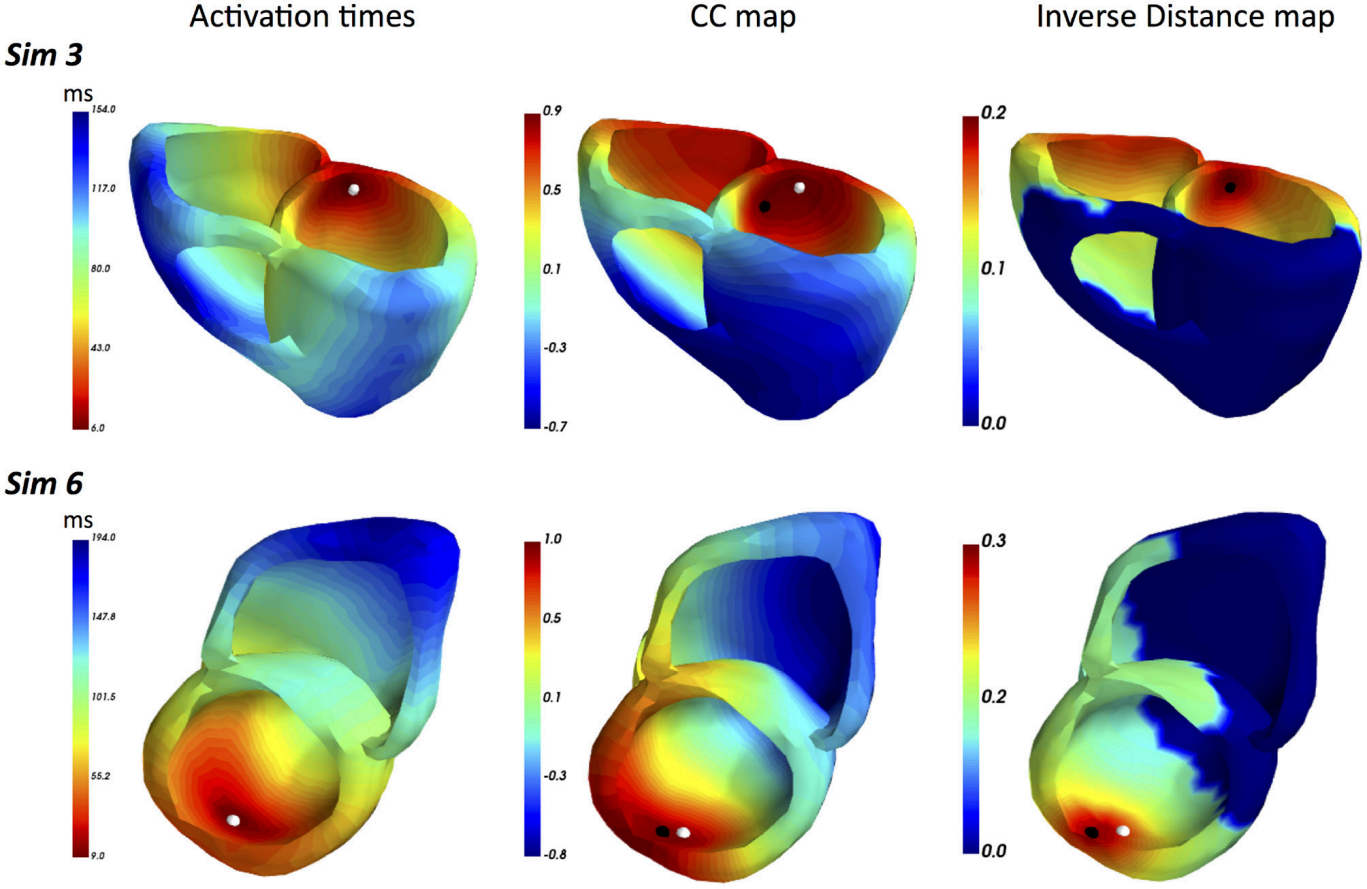
ECGI results for realistic simulated patterns 3 and 6. For pattern 3, the combination of FRA with subsequent time warping exhibited the highest error decrease from 15 to 0 mm. For this activation sequence, the onset reconstructed by FRA-DTW and marked by the black point coincides with the true focus. Pattern 6 was the only case among the considered eight, where the localization error provided by FRA-DTW was higher than that from the standard FRA.

A summary of LEs delivered by both FRA and FRA-DTW for the considered eight excitation patterns is shown in Table 1. The order of appearance is the same as in the original work (Janssen et al., 2018). As seen from the table, temporal alignment of the FRA-simulated test BSPMs resulted in lower LEs in seven cases. In Figure 3 shows the correlation maps obtained with FRA and inverse distance maps yielded from subsequent time warping for the excitation patterns 3 and 6. Although for pattern 6 the localization error from FRA-DTW is slightly higher than that of FRA, the focus was still correctly classified to originate from the endocardial wall.

**Table 1:** Localization error for the realistic ectopic simulations.

**Activation pattern**	**Localization error FRA, mm**	**Localization error FRA-DTW, mm**
Left side of septum (1)	0	0
Right side of septum (2)	11	7
Base LV near septum (3)	15	0
Base RV near septum (4)	18	4
LV epicardial free wall (5)	20	14
LV endocardial free wall (6)	12	19
RV endocardial free wall (7)	6	6
RV epicardial free wall (8)	14	7

### 3.2. ECGI of Single Pacings in CRT Patients

**Figure 4:**
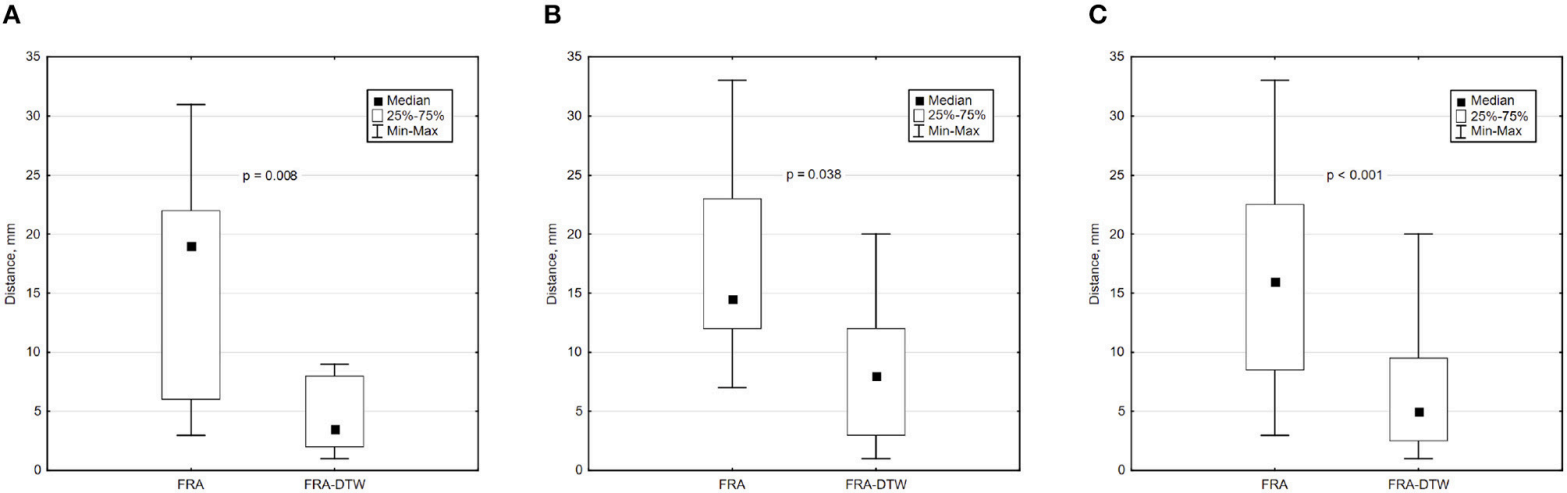
Box and whisker plots of LEs for FRA and FRA-DTW for the LV **(A)**, RV **(B)**, and all considered pacings **(C)**. The Wilcoxon signed-rank test was performed to compare localization accuracy provided by the two ECGI algorithms. A *p* < 0.05 was considered as statistically significant due to the relatively small sample size.

**Figure 5:**
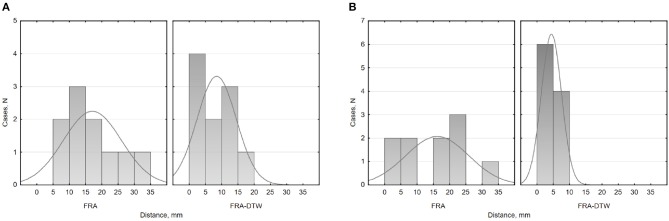
Histograms of LEs for the LV **(A)**, and RV **(B)** pacings. The curves represent fitted normal distributions.

**Figure 6:**
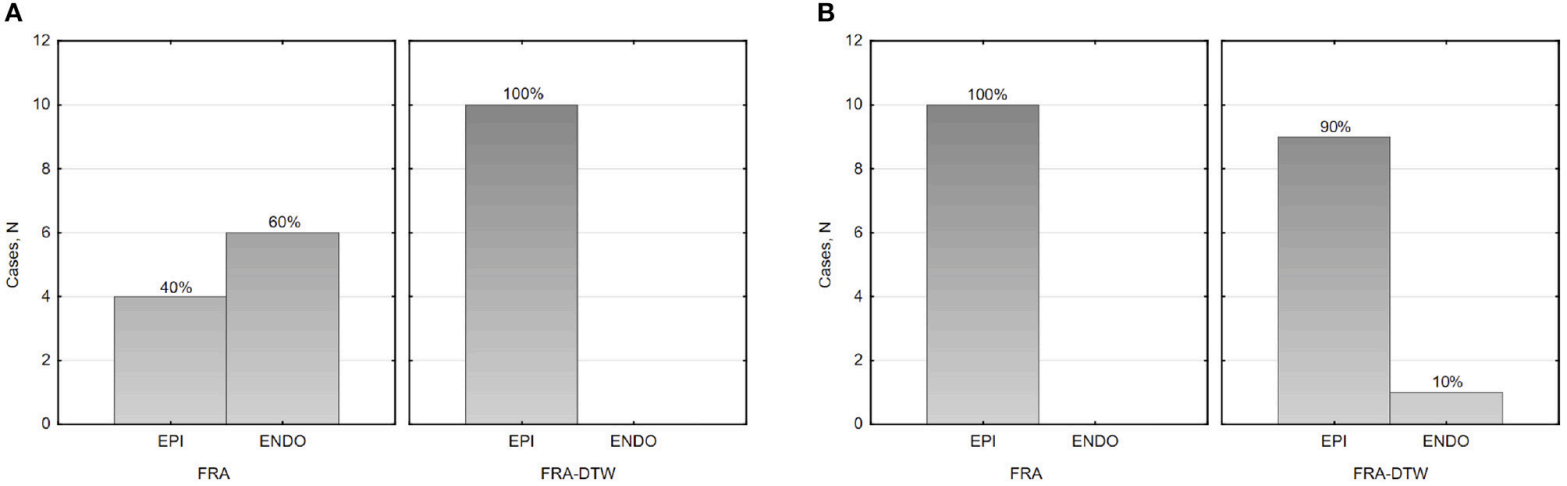
Histograms of the epi-endocardial early activation site's surface identification in the LV **(A)** and RV **(B)**.

**Figure 7:**
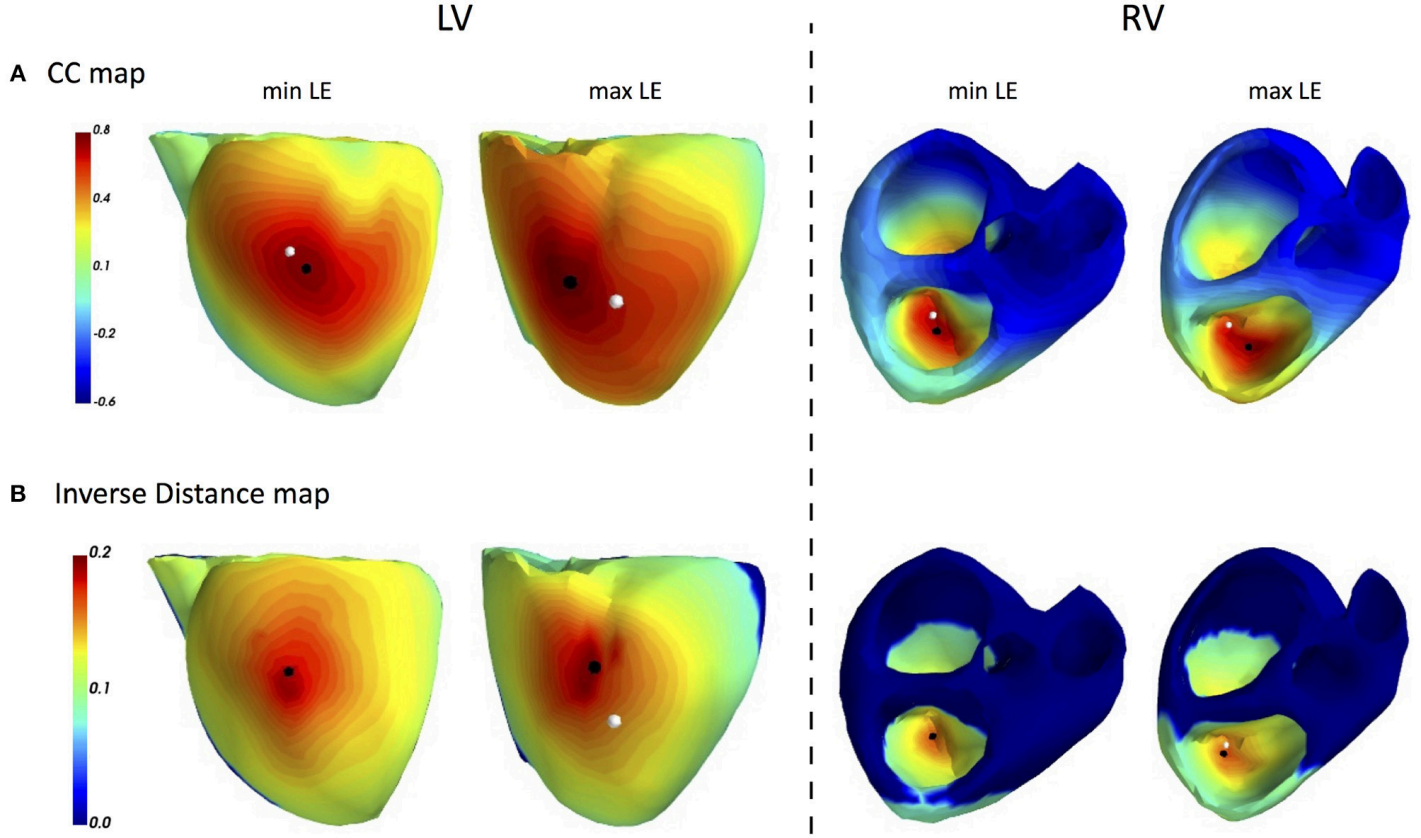
ECGI results for single pacings in CRT patients: FRA based CC maps **(A)** and FRA-DTW based inverse distance maps **(B)**. The white dot marks a pacemaker lead position projected on the cardiac surface, while the black point labels the reconstructed excitation origin. In the cases of minimal LEs for both LV and RV (first and third columns), the FRA-DTW method localized the onset at the same mesh node as the pacemaker lead. The accuracy of FRA was 8 and 16 mm, respectively. The cases, featuring maximal LEs for FRA-DTW, resulted in 16 and 16 mm for FRA.

The main accuracy characteristics are provided in Table 2. For all 10 patients, median (25–75% range) accuracy for FRA was 16 (8–23) mm and 5 (2–10) mm for FRA-DTW algorithm. The median accuracy for FRA in the LV was 15 (11–25) mm and 8 (3–13) mm for FRA-DTW algorithm, while in the RV the values were 19 (6–23) mm for FRA and 4 (2–8) mm for FRA-DTW. There was a significant difference in accuracy values calculated with FRA and FRA-DTW algorithms for LV, RV, and both LV and RV, which is shown in Figures 4A–C. It can also be seen from Figures 5A,B displaying the histograms that represent overall accuracy distributions for both algorithms. 95% bootstrap confidence intervals were also more narrow for LEs based on FRA-DTW compared to FRA algorithm. Furthermore, FRA-DTW was able to detect the correct (epi / endo) surface of an early activation for all LV pacings and one RV, whereas FRA detected the correct surface in four LV cases and wrongly associated all RV paced sequences to the epicaridal part of the heart surface (Figure 6). However, there was no significant difference in this accuracy feature between the RV and LV in every algorithm. For two patients, FRA-DTW resulted in a lower localization error in the RV septum compared to FRA. The LEs were reduced from 20–8 mm to 9–3 mm, respectively. Exemplarily, performance results from both methods for the cases featuring maximal and minimal LEs in the RV and LV are displayed in Figure 7.

**Table 2:** Main characteristics of ECGI accuracy for FRA and FRA-DTW algorithms. m, mean; SD, standard deviation; M, median; LQ, lower quartile; UQ, upper quartile; min, minimum; max, maximum, ratio of correctly detected early activation site's surface-R (in percents). For the localization error characteristics, 95% bootstrap confidence interval (CI) is provided in parentheses.

**Accuracy features, mm (95% bootstrap CI)**	**LV**		**RV**		**LV + RV**	
	**FRA**	**FRA-DTW**	**FRA**	**FRA-DTW**	**FRA**	**FRA-DTW**
m	17 (12–22)	8 (5–12)	16 (10–22)	4 (3–6)	17 (13–21)	6 (5–9)
SD	9 (3–11)	6 (4–8)	10 (6–12)	3 (2–4)	9 (7–11)	5 (3–7)
M	15 (12–23)	8 (3–13)	19 (6–24)	4 (2–8)	16 (10–22)	5 (3–9)
LQ (25%)	11 (7–15)	3 (1–9)	6 (3–20)	2 (1–4)	8 (2–14)	2 (1–4)
UQ (75%)	25 (14–33)	13 (7–20)	23 (18–31)	8 (4–9)	23 (18–31)	10 (6–14)
min	7	1	3	1	3	1
max	33	20	31	9	33	20
R	40	100	0	10	20	55

## 4. Discussion

In the presented work, the fastest route algorithm (FRA) was modified to account for local differences in conduction velocities. The dynamic time warping for matching simulated and measured BSPMs proved to increase robustness and accuracy of FRA. The improvements were shown both in simulation data as well as in a small CRT patient population with known inhomogeneous conduction velocity within the ventricular myocardium.

As within the standard FRA-based inverse procedure (van Dam et al., 2009b), for each cardiac mesh node activation sequences are generated with FRA, and the corresponding simulated test BSPMs are computed for a patient specific volume conductor model. Then, instead of correlation-based comparison between simulated and measured ECGs, the signals are temporally warped on the basis of an associated cost, or distance, function. To evaluate possible benefits of the warping step, we benchmarked this strategy against the CC-based FRA routine in three scenarios.

First, FRA-DTW was able to correct the localization error due to slower CV in a small heart region for a simple simulation case. Though this construct was purely artificial and did not represent a physiologically meaningful simulation, it served as a proof-of-concept that the excitation delays can be accounted for by BSPMs warping in the temporal domain.

In the next model-to-model comparison, the LEs were on average reduced by 5 mm when using the DTW-FRA algorithm vs. the standard FRA algorithm (Table 1). Previously, the authors examined three setups with respect to cardiac anisotropy used in the forward modeling: isotropic model, anisotropic model with equal anisotropy ratios in the extra- and intracellular spaces and an unequal anisotropy ratios case. For our purpose, we compared FRA to its warping modification only for the most realistic case of BSPMs produced by an anisotropic heart with unequal anisotropy ratios in the extra- and intracellualr domains. For seven cases, FRA-DTW performed similar or better than the original FRA. For a focus on the endocardial LV wall, FRA-DTW correctly identified endocardium as the onset origin, but resulted in a higher localization error compared to FRA. However, the ambiguity area, which can be taken as a region exhibiting the upper percentile of a similarity measure, was generally smaller for the reciprocal distance maps delivered by FRA-DTW method compared to the FRA-based CC maps.

Finally, we applied both methodologies to twenty paced activation sequences in ten CRT patients. The Wilcoxon non-parametric test suggested superior performance of FRA-DTW, yielding *p* = 0.0076 and 0.038 for LV and RV pacings, respectively. Importantly, the warping step improved localization accuracy for two cases with RV leads implanted in a septal area. An interesting aspect of all FRA related methods is the automatic detection of an early activation zone, which usually requires an additional sophisticated post-processing step for potential-based ECGI solutions (Duchateau et al., 2017). At the same time, both methods suffered from a low classification rate with respect to the correct cardiac surface for the RV pacings. We believe this issue to be due to the limited thickness of the RV wall, as no significant association was found between clinical characteristics, pacing modalities, number of body surface electrodes on one side and ECGI localization accuracy on the other. This fact can be considered as an indirect representativity evidence of the original clinical data set. We performed a bootstrap analysis for a robuster estimation of the obtained LEs because of a relatively small original sample size with the unknown distribution parameters in accordance to recommendations from Adèr (2008). In addition, bootstrap allowed us to use a resampling approach to mimic the process of obtaining new data sets, so that we can evaluate the variability of our assessment without generating additional samples. The bootstrap analysis showed that LE variability was significantly lower for the FRA-DTW algorithm, indicating its greater robustness against outliers. The bootstrap also helps to estimate LEs in the population, making results more predictable for clinical work. Furthermore, univentricular LV and RV pacings are the optimal ECGI validation data for single ectopic activation sequences. Thus, obtained results show the potential of the proposed methodology to significantly improve noninvasive detection of focal arrhythmia sources in clinical practice (Duchateau et al., 2018).

However, despite these promising results, it remains unclear how to relate an optimal warping path to the actual excitation in the heart. With this respect, we intend to perform further research by adding the optimization step in solving the NNLS problem using both initializations. FRA procedure in combination with time warping could compensate for the uniform excitation assumption of FRA. An example of explicit scar removal from the heart geometry for the EDL inverse model was presented by Oostendorp et al. (2002). Sapp et al. showed that the quality of potential-based ECG imaging of epicardial pacing sites in ventricular tachycardia patients deteriorates over myocardial scar or slowly conducting tissue (Sapp et al., 2012). Interestingly, the data from this study was recently reused by Zhou et al. investigating performance of a data-driven Bayesian method (Zhou et al., 2018). Though overall LEs were reduced by this novel approach, its accuracy was still suffering in cases when a pacing was performed in the scar region. These observations suggest potential improvements from combining ECGI in general, and FRA or other model-based approaches in particular, with anatomical substrate information.

Nonetheless, even in the absense of the underlying substrate data, ECGI was reported to provide important insights on the electrical excitation in CRT patients with varied LV pathology (Jia et al., 2006). A recent study by Bear *et al*. further demonstrated the ability of ECGI to accurately detect electrical dyssynchrony and identify the latest activation site with 9.1 ± 0.6 mm in Langendorff-perfused pig hearts (Bear et al., 2018). As a representative of model-based approaches, an offline created database of realistic forward simulations with different EP setups was shown to facilitate estimation of clinically relevant parameters, such as pacing configuration and CV profile (Giffard-Roisin et al., 2018). Such an offline strategy aiming at the real-time performance is computationally efficient, whilst enjoying an essential extensibility with every suitable clinical case. Our future efforts will be focused on deploying imaging modalities together with personalized biophysical computer models and ECG imaging.

## 5. Conclusions

In this work, we quantitatively assessed FRA performance on CRT patients. An important enhancement of the FRA method, a temporal warping of FRA-generated BSPMs sequences, was introduced. Using FRA-DTRW reduced the LE by approximately a factor of two, demonstrating a significant accuracy improvement for clinical data of CRT patients with a complex etiology.

## 6. Limitations

Evaluation of ECGI accuracy using CRT devices is intrinsically limited to the LV lateral wall, RV apex and septum, while other anatomical regions cannot be tested in the same manner. For drawing clinically relevant conclusions, another study with a larger sample size should be considered for a detailed representativity evaluation of the data used. The presented bootstrap analysis models potential outcomes of such a study and, therefore, serves as a reference point for future investigations.

The lack of late gadolinium enhancement MRI data in patients with previous myocardial infarction did not allow us to quantify the influence of this factor on the tested algorithms.

## Data Availability

The datasets for this study will not be made publicly available because the clinical datasets belong to the Almazov National Medical Research Center, Saint-Petersburg, Russia. Therefore, the corresponding author is not authorised to provide them upon request. However, the respective co-authors can be asked for it.

## Author Contributions

DP conceived the presented warping extension of FRA, designed the simulation setups and conducted the numerical part of the clinical study. MC performed statistical analysis of the clinical results. PvD provided technical expertise and revised the methodology. DP, MC, and PvD discussed the main findings and results, and wrote the manuscript. MC, SZ, and MB conducted the clinical data acquisition and post-processing. TT and DL provided medical expertise and contributed to the clinical study design.

### Conflict of Interest Statement

DP and MC are employed by EP Solutions SA, Switzerland (EPS). SZ and MB are providing clinical support for EPS. PvD is an owner of Peacs BV, Netherlands. The remaining authors declare that the research was conducted in the absence of any commercial or financial relationships that could be construed as a potential conflict of interest.

## A. Appendix: Pseudo-Code for Employed Pipeline

Some datasets of ventricular ectopic excitation, though simulated with a cellular automaton, could be found on the open ECGI validation platform EDGAR. For dynamic time warping, the authors used a *Python* implementation available under https://github.com/pierre-rouanet/dtw. The fastest route algorithm is based on Dijkstra's shortest path routine, for which multiple numeric realizations are freely available.

To systematize the inverse processing flow applied, we provide its pseudo-code in the following:

**Table:** Algorithm 1^*^

Inverse distance map 1/*c*_*p*_ calculation
**Input:** BSPMs matrix *Y*, transfer matrix *A*, functions *FRA* and
*DTW*
[*m, n*] = *size*(*A*)
*c*_*p*_ = *zeros*(*n*,)
*Y* = (*Y*−*mean*(*Y, axis* = 0))/*std*(*Y, axis* = 0)
*z*-normalization of each BSPMs column
**for** *i* = 1, …, *n* **do**
τ_*i*_←*FRA*(*i*) FRA-based activation times due to the onset
at the cardiac node *i*
$\tilde{Y}=A*TMP\left(\tau_{i}\right)$ test BSPMs due to cardiac node *i* using
formula (1)
$\tilde{Y}=\left(\tilde{Y}-mean\left(\tilde{Y},axis=0\right)\right)/std\left(\tilde{Y},axis=0\right)$
$dist_{i},p_{i}\leftarrow DTW\left(\tilde{Y},Y\right)$
the optimal path *p*_*i*_ itself is not further used
*c*_*p*_(*i*) = *dist*_*i*_
**end for**
**return** 1/*c*_*p*_
